# Dirac semimetal thin films in in-plane magnetic fields

**DOI:** 10.1038/srep34882

**Published:** 2016-10-10

**Authors:** Zhuo Bin Siu, Mansoor B. A. Jalil, Seng Ghee Tan

**Affiliations:** 1 Computational Nanoelectronics and Nanodevices Laboratory, National University of Singapore, Singapore; 2 Data Storage Institute, Agency for Science, Reseach and Technology, Singapore

## Abstract

In this work we study the effects of in-plane magnetic fields on thin films of the Dirac Semimetal (DSM) Na_3_Bi where one of the in-plane directions is perpendicular to the *k*-separation between the two Weyl nodes that exist for each spin orientation. We show numerically that the states localized near the surfaces of these thin films are related to the Fermi arc states in semi-infinite slabs. Due to the anisotropy between the two in-plane directions, the application of a magnetic field along these directions have differing effects. A field parallel to the *k* space separation between the Weyl nodes leads to a broadening of the surface state band and the formation of an energy plateau, while a perpendicular field shifts the energy where the hole and particle bands meet upwards, and sharpens the tips of the bands. We illustrate the effects of these changes to the dispersion relation by studying the transmission from a source segment without a magnetic field to a drain segment with a field, with the field and interface at various in-plane directions.

The Dirac Semimetal (DSM)^1^,^2^,^3^,^4^ is a recently discovered topologically non-trivial state which has attracted much attention. Like the surface states of the more established three-dimensional topological insulators (TI)^5^,^6^, the low energy spectrum of DSMs take the form of Dirac cones. Unlike the Dirac cones in three-dimensional TI surface states which have linear dispersion only in the two dimensions in the plane of a TI *surface*, the DSM Dirac cones disperse linearly in all three *k*-space dimensions in *bulk* DSMs about the Dirac points. Moreover, unlike in conventional TIs where there is a odd number of Weyl nodes, the Weyl nodes in DSMs occur in pairs with one member of each pair acting as a Berry curvature source and the other member acting as a Berry curvature sink. These pairs of Weyl nodes in DSMs give rise to surface states in the form of Fermi arcs linking the two points in *k*-space when the bulk DSM is truncated perpendicular to the direction of the *k*-space separation between the Dirac points. These Weyl nodes are topologically stable against perturbations which preserve the translational symmetry. To date, two materials, Cd_3_As_2_^7^,^8^,^9^,^10^ and Na_3_Bi^11^,^12^,^13^ have been experimentally confirmed to host the DSM state.

The effects of electric fields applied perpendicular to the surfaces of quasi-two dimensional thin films and quasi-one dimensional Na_3_Bi nanowires have been studied recently^14^,^15^. Whereas the effects of magnetic fields on DSMs have been studied previously (for example, in refs 16, 17, 18), these works have tended to focus on magnetic fields perpendicular to the plane of DSM slabs and thin films. In this work, we study the effects of *in plane magnetic fields* on DSM thin films. To lay the foundations for our subsequent discussion, we first review the energy dispersion of bulk Na_3_Bi and the emergence of Fermi arcs on semi-infinite slabs of Na_3_Bi terminated perpendicular to the *k*-space separation between the Dirac points. We then consider DSM thin films with finite thickness, and next move on to discuss the effects of in-plane magnetic fields on the dispersion relations. We finally showcase one consequence of the magnetic fields by considering the transmission from a source DSM thin film segment without a magnetic field to a drain DSM segment with a magnetic field for various directions of magnetic fields and source-drain interfaces. The anisotropy between the directions parallel and perpendicular to the *k* space separation between the Dirac points gives rise to a rich transmission profile.

## Bulk eigenstates

The Hamiltonian for bulk Na_3_Bi reads


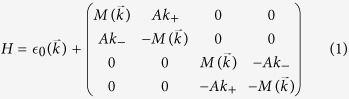


where 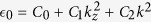 and 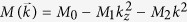
1,2, 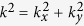. *A*, *C*_0_, *C*_1_, *C*_2_, *M*_0_, *M*_1_ and *M*_2_ are material parameters for which we used the values in ref. 1. The Hamiltonian consists of two uncoupled blocks representing the spin up and spin down states, which we can consider separately. We focus on the spin up states.

Diagonalizing the spin up block yields the eigenvalues


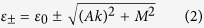


and the (unnormalized) eigenspinors


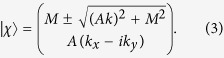


**Figure 1:**
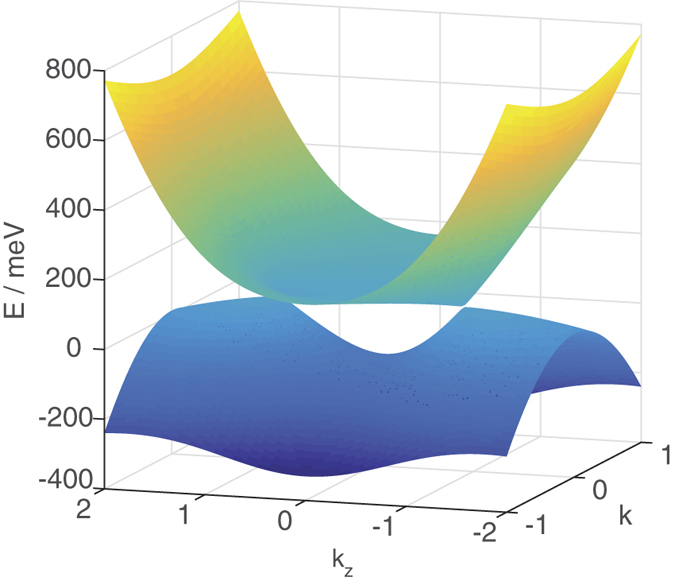
Energy offset from *ε*_0_ plotted as a function of 
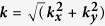
 and *k*_*z*_.

The bulk energy dispersion is shown in Fig. 1. The Hamiltonian is isotropic in the (*k*_*x*_, *k*_*y*_) plane and thus we have set one of the axes in Fig. 1 as 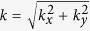. The dispersion relation consists of two parabolic cone-like structures above (below) *ε*_0_ corresponding to the +(−) sign of Eq. 2 with the cone tips squashed inwards to form cusps, so that the points where the cone tip starts curving inwards correspond to the lowest (highest) energy at the two Dirac points. The two Dirac points have energy *ε*_0_ and lie along the *k*_*x*_ = *k*_*y*_ = 0 line.

## Semi-infinite Slabs

**Figure 2:**
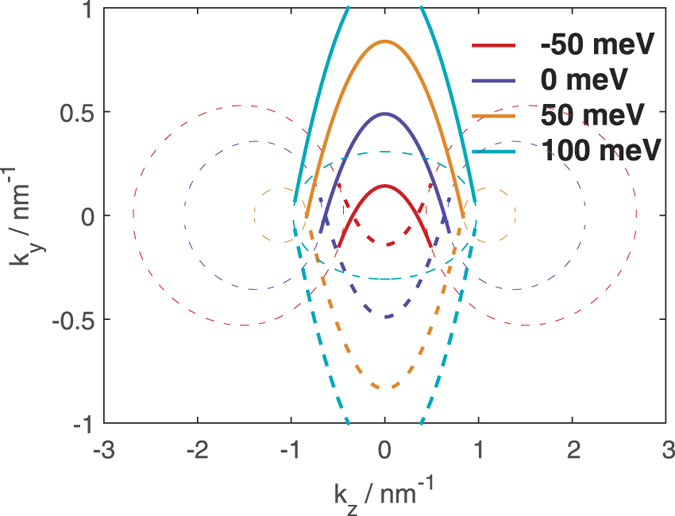
The thick lines indicate the Fermi arcs at the values of energy indicated in the legend. Solid lines correspond to the spin up Fermi arcs at a +*x* terminated surface on a semi-infinite slab extending to *x* → −∞ while dotted lines correspond to arcs on *x* terminated surfaces extending to *z* → +∞. The thin dotted lines are the bulk EECs for an infinite slab at *k*_*x*_ = 0 at the indicated values of energy.

One hallmark of a DSM is the emergence of Fermi arcs when a bulk DSM is truncated perpendicular to the *k*-space separation between the Dirac points. We therefore consider a semi-infinite slab terminated at the *x* direction so that the slab extends to infinity along the *y* and *z* directions, and *k*_*y*_ and *k*_*z*_ are good quantum numbers. Relegating the details of the calculation to Sect. I of the Supplementary Information, we present in Fig. 2 the calculated Fermi arcs for spin up at various values of energy for semi-infinite slabs terminated along the *x* = 0 line and extending to *x* → ±∞ respectively. We also show the *infinite* bulk equal energy contours (EECs) at *k*_*x*_ = 0 at those values of energy. We stress that the calculation of the Fermi arcs for the *semi*-infinite slabs, and that of the EECs for the infinite bulk are two separate calculations. For the infinite bulk, the EECs were obtained by searching for values of *k*_*y*_ and *k*_*z*_ satisfying Eq. 2 for the given value of energy with *k*_*x*_ set explicitly to 0. For the semi-infinite system, the Fermi arcs were obtained by searching for values of *k*_*y*_ and *k*_*z*_ satisfying Eq. 2 where the corresponding eigenstate wavefunctions vanish at *x* = 0 as detailed in the Supplementary Information.

The spin up Fermi arcs for semi-infinite slabs extending to *x* → ±inf are reflections of each other along the *k*_*y*_ = 0 line. (The spin down Fermi arcs are the reversal of the spin up Fermi arcs).

The arcs emerge once the energy is increased from −∞ to the bottom of the bulk hole band cusp and continue to exist as the energy is increased towards +∞. The arcs are tangential to the bulk EECs calculated with the wavevector perpendicular to the surface (*k*_*x*_ in this case) set to 0. (The two circles, one on the left and the other on the right, for each of the EECs for the three lower values of energies are the cross sections on the two ‘humps’ on either side of the cusp in the hole bands. The single circle in the middle for *E* = 100 meV is the cross section of the ‘cone’ of the bulk particle band. This value of energy lies above the top of the cusp of the bulk particle band).

We shall later see that the arcs play a significant role in the dispersion relations of thin films.

## Thin films

We next consider thin films of infinite dimensions along the *y* and *z* direction, and finite thickness along the *x* direction. *k*_*y*_ and *k*_*z*_ are hence good quantum numbers. In contrast to the semi-infinite slab which has only a single surface, a thin film has both an upper and a lower surface. As a result we expect to see aspects which we are familiar with from the previous discussion on semi-infinite slabs, as well as features which now emerge because of the finite thickness.

We solve the eigenspectrum of the thin film numerically. We adopt the hard-wall boundary conditions under which the wavefunctions vanish at *x* = 0 and *x* = *W*, *W* being the thickness of the film. Under these boundary conditions, the spatial part of the eigenstate wavefunctions can be expanded as a linear combination of the normalized eigenstates of the infinite potential well |*ϕ*_*n*_〉 where 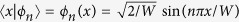. Since the spin up and spin down states are decoupled in the Hamiltonian, we consider each spin separately. For each spin and given value of *k*_*y*_ and *k*_*z*_ the relevant part of the Hamiltonian *H*(*k*_*y*_, *k*_*z*_)_±_ (the ± subscript referring to the spin up/down parts of the Hamiltonian) can be expanded in the basis of the |*ϕ*_*n*_〉 states. This gives a a numerical matrix 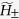 with matrix elements 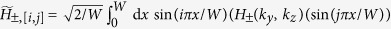. The matrix can then be diagonalized numerically to obtain the eigenenergies and eigenstates.

**Figure 3:**
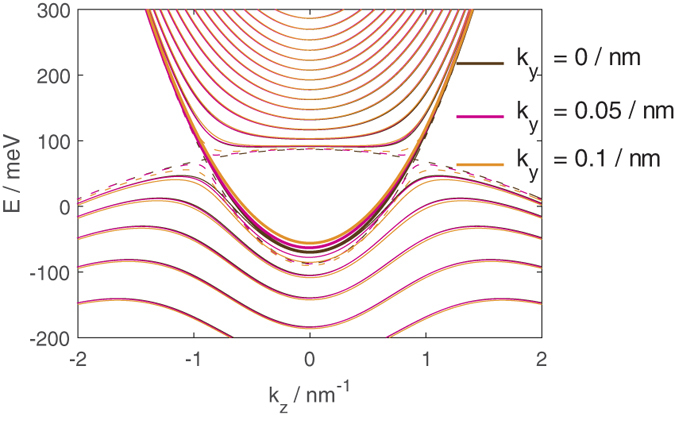
The dispersion relations for a 20 nm thick film as a function of *k*_*z*_ for various values of *k*_*y*_ indicated by the colors of the lines. The dotted lines show the bulk energy values assuming *k*_*x*_ = 0. The thicker lines denote what we shall, for brevity, refer to as the ‘lowest energy particle band’ (LEPB).

Figure 3 shows the dispersion relations for a 20 nm thick film at various values of *k*_*y*_ as a function of *k*_*z*_.

Similar to the semi-infinite slab, the hole bands bend downwards in energy at small |*k*_*z*_| to give rise to a cusp between the two Dirac points. The finite thickness of the thin film here and leads to the formation of subbands.

There is a subband, highlighted with slightly thicker line Fig. 3, which traces the bulk *k*_*x*_ = 0 particle band for *k*_*z*_ values outside the bulk cusp and the bulk hole band inside the bulk cusp. This band is special, as we shall explain later. For brevity we shall refer to it as the Lowest Energy Particle Band (LEPB), and the subband immediately below it in energy as the Highest Energy Hole Band (HEHB). Except for the LEPB, the particle band bottoms are bounded in energy above the *k*_*x*_ = 0 bulk particle band. The energies of the hole bands at |*k*_*z*_| lying outside the two bulk Dirac points are also bounded below the *k*_*x*_ = 0 bulk hole band. At energies above the Dirac point corresponding to |*k*_*z*_| lying outside the two bulk Dirac points, the energy of the LEPB follows that of the bulk particle states closely. At energies below the Dirac point the energy of the lowest energy particle states lie slightly above the top of the ‘cusp’ in the hole energy bands for *k*_*z*_ lying between the two Dirac points. The band bottoms (tops) of the particle (hole) bands increase (decrease) in energy monotonously with |*k*_*y*_|.

**Figure 4:**
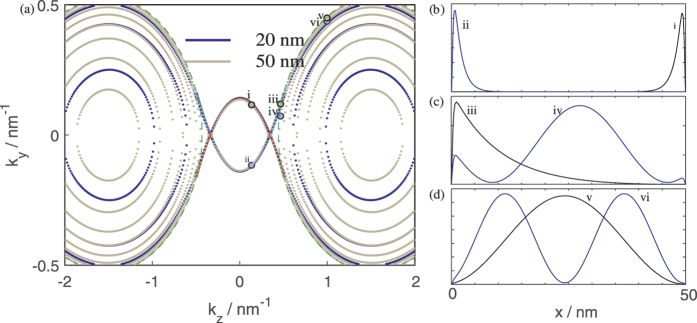
In panel (a) on the left, the thick solid lines around the *k*-space origin represent the bulk Fermi arcs for slabs infinite in the *y* and *z* direction, and semi-infinite in the +*x* and −*x* direction at *E* = −50 meV. The dotted green lines are the bulk EECs at the same value of energy, while the dots trace out the EECs for a 20 nm, and 50 nm thick film as indicated by the color of the dots. The relative charge densities of the states within the thickness of the thin film at various points in *k*-space and *E* = −50 eV marked *i* to *vi* on the EECs are plotted out in panels (b) to (d).

For another perspective we now plot out the *k* space EECs for the 20 nm thick thin film which we have been considering, as well at that of a thicker 50 nm thick film at *E* = −50 meV in Fig. 4. This is a value in energy which lies above the bottom of the LEPB but below the two ‘humps’ surrounding the small |*k*_*z*_| cusp.

One dominant feature of the figure is the presence of two series of concentric rings of EECs, one series of rings on the left and the other series on the right which we shall for simplicity refer to as the left and right onions. These two onions correspond to the two ‘humps’ in Fig. 3 that, at higher energies, eventually form the two Dirac points while the empty space in between corresponds to the cusp between the two humps in Fig. 3. The outermost rings of each onion correspond to the HEHB. The states that lie outside the two bulk Fermi arcs are bounded by the bulk EECs indicated by the green dotted lines. These states, such as states (iv) to (vi) in the diagram, are bulk states where there is significant charge density away from the two boundaries. In particular, away from the Fermi arcs, the bulk states (v) and (vi) resemble the usual infinite quantum well states with increasing number of density nodes as we move into the interior of the onion. As one would expect from the infinite quantum well, a larger thickness results in a larger number of states at a given energy.

We now focus on the *k*-space around the vicinity of the bulk Fermi arcs. The bulk Fermi arcs for the upper and lower surface ‘penetrate’ each other so that for fixed value of |*k*_*y*_| < 0.15 nm^−1^ a line of constant *k*_*z*_ cuts through both the upper and lower surface bulk Fermi arcs. There are always thin film states which lie near the bulk Fermi arcs regardless of the thickness of the film. The states which lie near the Fermi arcs are localized near the surface (see the states labeled (i) to (iii)) which the arcs correspond to, although the extent of localization decreases with increasing |*k*_*z*_| (compare state (iii) with states (i) and (ii)).

States (i) and (iii) share approximately the same *k*_*y*_ value. Point (i) with the smaller |*k*_*z*_| lies on the LEPB and point (iii) with the bigger |*k*_*z*_| lies on the HEHB. The states lying in the vicinity of the bulk Fermi arcs for small |*k*_*z*_| smaller than the |*k*_*z*_| which the two Fermi arcs intersect each other lie on the LEPB, while the states that lie on the Fermi arcs for larger values of |*k*_*z*_| lie on the HEHB. The states on the LEPB are therefore always surface states. The states on the HEHB (i.e. the outermost rings of the onions) for small |*k*_*z*_| lying on the Fermi arcs are also surface states (e.g. iii), but those states on the HEHB with larger values of |*k*_*z*_| (e.g. vi) are not surface states.

An examination of the EECs and density distribution at other values of energies indicates that the LEPB states at a given energy are always located near the bulk Fermi arcs and are localized near either the upper or lower surface, whereas the other states present at the same value of energy located away from the bulk Fermi arcs are bulk states. (We show some of these LEPB states in Supp. Fig. 1 in the Supplementary Information file). The *k*-space locations of the LEPB states at a given energy are only very weakly dependent on the film thickness. Together, these indicate that the LEPB states originate from the bulk Fermi arcs and may share the topological protection of the latter. This robustness makes the LEPB states of particular interest in potential device applications. We shall consequently concentrate on the low energy regime in which only the LEPB is present.

## Effects of an in-plane Magnetic Field

The effects of an out-of-plane electric field on a DSM thin film/quasi-one-dimensional nanostructure were investigated in refs 14 and 15. Here we investigate how a magnetic field in the *y* and *z* directions affect the dispersion relations. The modification of the equal energy contours by an in-plane magnetic field, and the anisotropy between the *k*-space directions perpendicular and parallel to the *k* space separation between the Dirac points shall, as we show in the next section, lead to dramatic differences in the transmission from a source DSM segment without a magnetic field to a drain segment with a field as the energy and magnetic field are varied.

We model a magnetic field in the *y* direction via the canonical substitution 
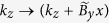
 and a field in the *z* direction via 
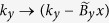
 where we have absorbed the various physical constants into 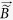. In our numerical calculations the units for *k*_*y*_ are in nm^−1^, so that the quantities of 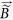 which appear subsequently carry units of nm^−2^. (One nm^−2^ of 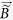 corresponds to 660 T).

**Figure 5:**
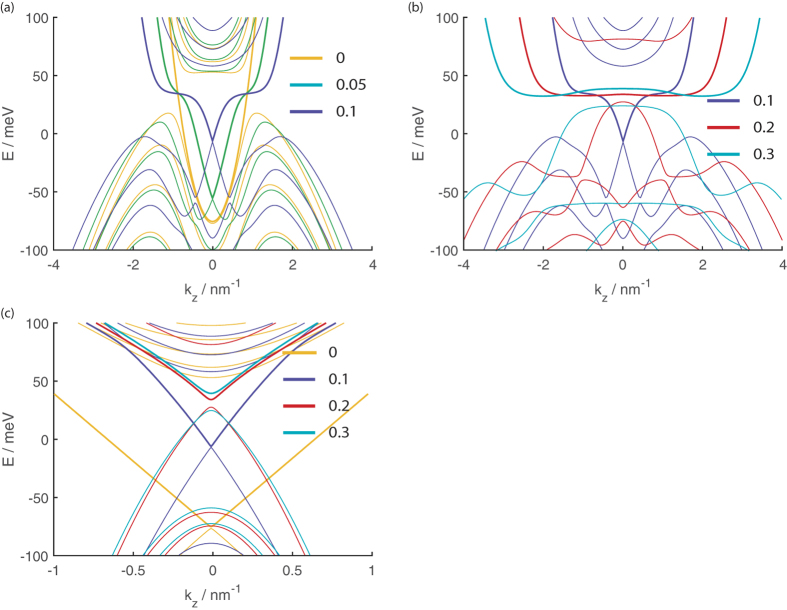
The dispersion relations for a 20 nm thick slab (**a**,**b**) as a function of *k*_*z*_ at *k*_*y*_ = 0, and (**c**) as a function of *k*_*y*_ at *k*_*z*_ = 0 at the values of 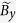 indicated in the figure legends. The LEPBs are indicated by thicker lines.

Figure 5 shows the dispersion relations calculated as functions of the *y* and *z* wavevectors with the wavevector in the other direction set to 0 at various values of magnetic field in the *y* direction. The *y* direction is perpendicular to the *k*-space separation between the two Dirac points. We have split the *k*_*y*_ dispersion relations into two separate panels in the figure for clarity to avoid having too many lines in a single panel.

Focusing first on panel (a) of the figure, we see that a small *B*_*y*_ leads to the HEHB states with small |*k*_*z*_| bending upwards in energy to form a small upwards pointing kink centered at *k*_*z*_ = 0. The band bottom of the LEPB band sharpens to form a downwards pointing kink at *k*_*z*_ = 0 so that the *k* space vicinity around where the LEPB and HEHB touch at 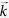 = 0 is now reminiscent of a Dirac cone. The portion of the band on the left and right side of this downwards kink form a plateau at around 60 eV in energy. The “Dirac point” where the LEPB and HEHB touch shifts upwards in energy as *B*_*y*_ increases until it hits the band plateau (see panel (b)) at around 60 eV. At this stage the downwards pointing kink in the particle band bottom disappears into the plateau. As *B*_*y*_ increases even further the middle of the LEPB ‘plateau’ becomes concave, the upwards kink of the HEHB blunts out and an energy gap between the LEPB and the HEBB opens up. Panel (c) of the figure shows that in contrast to the complicated dispersion relation along the *k*_*z*_ direction, the energy of the LEPB increases monotonically with |*k*_*y*_|. We shall show the EECs at various values of energy and 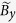 in the next section when we discuss the transmission from a source segment without magnetic field to a drain segment with a magnetic field.

**Figure 6:**
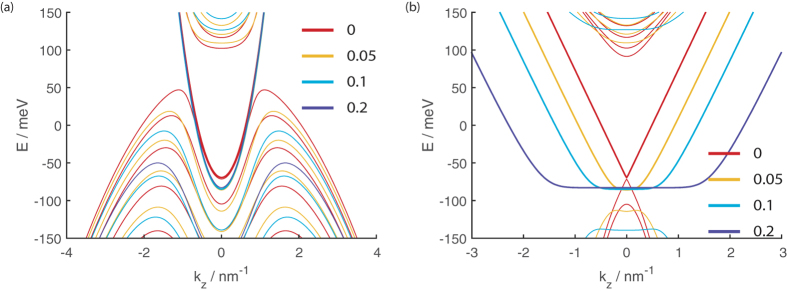
The dispersion relations for a 20 nm thick slab for (**a**) as a function of *k*_*z*_ at *k*_*y*_ = 0, and (**b**) as a function of *k*_*y*_ at *k*_*z*_ = 0 at the values of 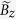 indicated in the figure legends. The LEPBs are indicated by thicker lines.

Figure 6 shows the dispersion relations when a magnetic field in the *z* direction parallel to the *k* space separation between the two Weyl nodes is applied.

Panel (a) shows that the dispersion relation of the LEPB along the *k*_*z*_ direction is not very much affected by the magnetic field. A larger field however does push the other particle bands upwards in energy, and the hole bands downwards in energy, as well as increases the energy separation between the subbands. The *z* magnetic field has a larger effect on the dispersion relation along the *k*_*y*_ direction. As the magnetic field is increased, the band bottom of the LEPB flattens out and forms a plateau for an increasing range of |*k*_*y*_|.

## Transmission

Here we consider the transmission of the LEPB states from a source DSM film segment without a magnetic field, to a drain segment with a magnetic field. We consider the cases where the interface between the two segments lies along either *y* = 0 or *z* = 0 line so that the source and drain segments have semi-infinite extents in the in-plane (*yz*) direction perpendicular to the interface and infinite extent parallel to it. The transverse momentum parallel to the interface direction is hence a good quantum number and is conserved between the two segments.

We briefly outline how we calculated the transmission for the case where the interface between the source and drain lies along *y* = 0. (The calculation for an interface along *x* = 0 follows analogously.) In this case *k*_*z*_ is conserved. For each given value of energy and each given value of *k*_*z*_, we obtain the eigenstates with the given values of energy and *k*_*z*_ in both the source and drain segments numerically using the infinite potential well |*ϕ*_*n*_〉 representation of the Hamiltonian outlined at the beginning of the previous section. We then pick out those source eigenstates which propagate in the +*y* direction from the source to the drain, i.e. the states for which −*i*〈[*y*, *H*]〉 > 0. Consider the *a*th source state on the source drain interface which we denote as 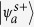 where the *s*+ superscript denotes that this is a *s*ource state propagating in the +*y* direction and the *a* subscript indexes the state. The scattering state with this *a*th state incident on the interface, |Ψ_*a*_〉, is a linear combination of the incident state, the states reflected back to the source, and the states transmitted across the interface 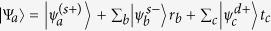 where the (*s*/*d*) in the superscript indicates if these are source or drain states, the ± the direction in which these states propagate or decay, and the *a*, *b* and *c* subscripts label the states. The reflection coefficients *r*_*b*_ and transmission coefficients *t*_*c*_ can then be solved for numerically by matching the wavefunctions and their first and second *y* derivatives on both sides of the interface. The resulting current transmitted across the interface can then be calculated. The net transmission is then obtained by integrating over all the source states on the EEC at the given energy propagating in the +*y* direction.

Since the integration over all the source states contributing to the transmission at a given energy is essentially an integration over the transverse momentum range spanned by these states, the transmission is very much affected by the overlap between the ranges of transverse momentum spanned by the source and drain states.

The anisotropy between the *k*-space directions parallel (*z*) and perpendicular to the *k*-space separation leads to different transmission profiles depending on the direction of the interface.

**Figure 7:**
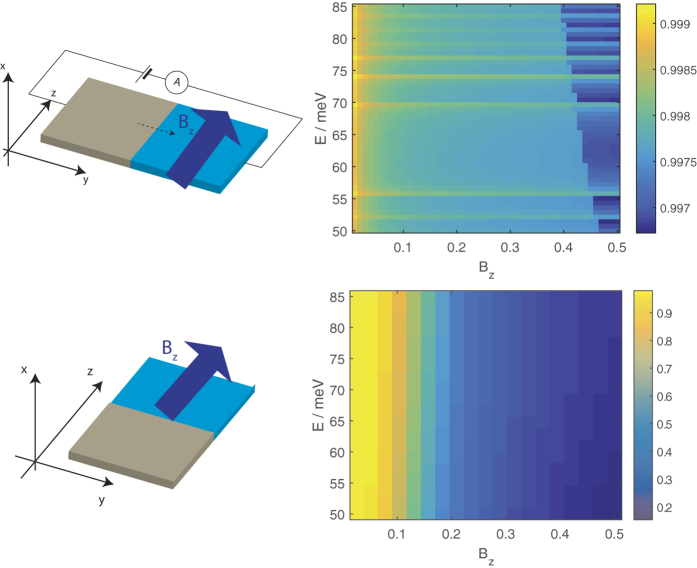
The transmission from a source segment of 20 nm thick DSM thin film with no magnetic field to a drain segment with a magnetic field in the *z* direction for (top) an interface parallel to the *z* direction and (bottom) an interface parallel to the *y* direction.

Figure 7 shows the transmission profiles from a source segment without a magnetic field to a drain segment with a magnetic field in the *z* direction. (The energy range is chosen so that the only propagating states in the source are the LEPB states. The lower panel of Fig. 7 corresponds to the case where the electromagnetic vector gauge potential is 
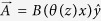
. This gives, in addition to the step-function variation of *B*_*z*_ across the source-drain interface, an additional 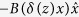 magnetic field not depicted in the figure. This additional field preserves 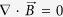 at the interface). The transmission for an interface parallel to the *z* direction is almost unity and exhibits a decrease with magnetic field for an interface parallel to the *y* direction.

**Figure 8:**
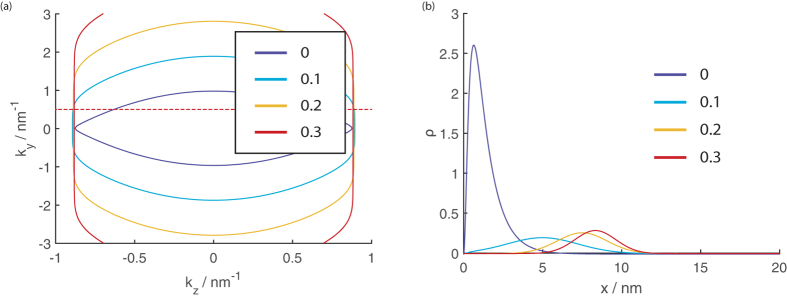
(**a**) The EECs for a 20 nm thick DSM film at 70 eV at various strengths of *B*_*z*_ indicated in the legend. (**b**) The particle density profiles within the thickness of the thin film at various values of *B*_*z*_ at *k*_*y*_ = 0.5 nm^−1^ as indicated by the dotted line on the EEC plots on panel (a).

The reason for these trends can be found in the EECs of the source and drain segments shown in Fig. 8.

The *k*_*y*_ range spanned by the EECs increases with *B*_*z*_ while the *k*_*z*_ range spanned remains constant. For an interface along the *z* direction (top of Fig. 7) we essentially integrate across the *k*_*z*_ range spanned by the source states in order to calculate the transmission. At any given value of *k*_*z*_ the profile of the source and drain EECs have a similar trend of curving outwards in the *k*_*y*_ direction. This, and the fact that the *k*_*z*_ range spanned by the source and drain EECs are almost the same and that the EECs have the same contour which bows outwards along the *k*_*y*_ direction contribute towards the large transmission. For an interface along the *y* direction, we integrate along the *k*_*y*_ range spanned by the source state. Although the *k*_*y*_ range spanned by the drain EECs completely overlaps that spanned by the source EEC, the profile of the EEC at a given *k*_*y*_ between the source and drain segments are quite different–the source EEC (where *B*_*z*_ = 0) curves outwards along the *k*_*y*_ direction but the drain EECs flatten out with increasing *B*_*z*_. This flattening out of the drain EECs is accompanied by a shift in the position along the thickness of the film (in the *x* direction) where the charge carriers with group velocity from the source to the drain are concentrated. Panel (b) in the figure shows that at low fields the charge carriers are localized near the boundaries of the film, but they increasingly get shifted towards the interior of the film with increasing *B*_*z*_. This reduces the overlap between the wavefunctions of the LEPB states in the source and drain segments and leads to the trend of decreasing transmission with *B*_*z*_ at a given energy.

**Figure 9:**
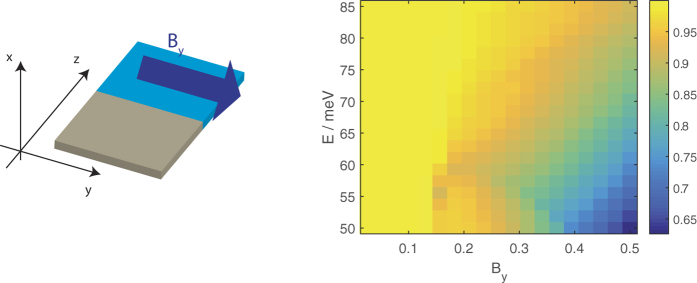
The transmission from a source segment without a magnetic field to a drain segment with a magnetic field in the *y* direction for an interface parallel to the *y* direction.

We now shift our focus to the effects of applying a magnetic field in the *y* direction. Figure 9 shows that the transmission from a source segment to a drain segment with a magnetic field in the *y* direction for an interface parallel to the *y* direction decreases with increasing magnetic field.

**Figure 10:**
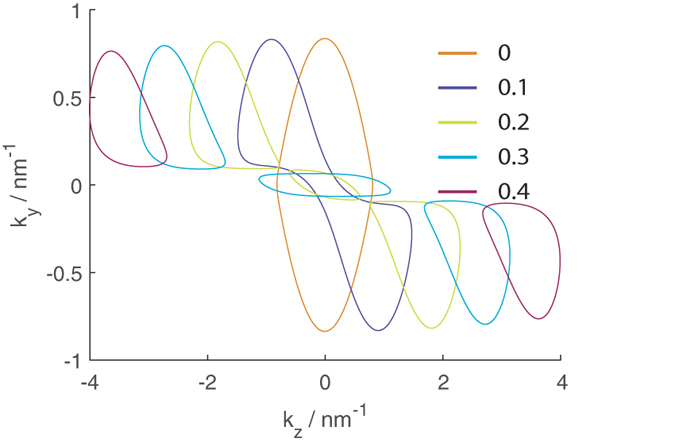
The EECs at *E* = 50 meV at various values of 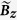 indicated in the legend.

This can be explained from the shapes of the EECs as shown in Fig. 10. Comparing against Fig. 5, the single lobe for 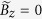 corresponds to the cross section of the LEPB ‘cone’. The small, narrow ‘waist’ of the 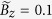 around 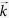 = 0 corresponds to the cross section of the downwards pointing kink in the LEPB. The central lodes enclosing 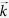 = 0 surrounded by side lobes for 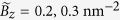 are the cross sections of the HEHB. There is no central lobe in the 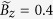 because the top of the HEHB has fallen below *E* = 50 meV.

Calculating the transmission across an interface parallel to the *y* direction involves integrating over the *k*_*y*_ range spanned by the source EEC. The *k*_*y*_ range spanned by the drain EECs decreases with increasing *B*_*y*_. As 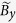 increases from zero, gaps in the EECs begin to open apart so that the EEC which, is originally a single closed curve at *B*_*y*_ = 0, breaks into three separate curves at 
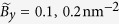
 in the figure. The gaps in *k*-space between the EEC curves represents an absence of propagating states at those values of *k*_*y*_ and lead to a drop in the transmission. Once 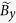 increases beyond around 0.2 where the energy at 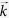 = 0 of the LEPB rises above the energy at finite |
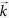
|, the band top of the HEHB drops in energy with increasing 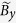. An energy gap opens up. This is manifested as the absence of the central EEC lobe at the larger values of 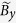 in the figure. At the same time, the *k*_*y*_ ranges spanned by the side lobes decrease with 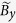. All of these contribute to the drop in transmission with 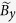.

**Figure 11:**
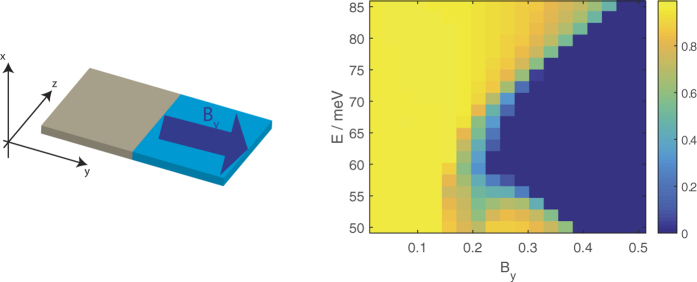
The transmission from a source segment without a magnetic field to a drain segment with a magnetic field in the *y* direction for an interface parallel to the *z* direction.

Finally, Fig. 11 shows the transmission from a source segment without a magnetic field to a drain segment with a magnetic field in the *y* direction with the interface parallel to the *z* direction.

The decrease in transmission at a given value of energy with *B*_*y*_ can also be explained by the EEC profile in Fig. 10. Here since the interface is parallel to the *z* direction *k*_*z*_ is conserved. From the figure it is evident that as *B*_*y*_ is increased the EECs of the drain states are shifted towards larger |*k*_*z*_| so that the *k*_*z*_ overlap between the source EEC centered around *k*_*z*_ = 0 and the drain EECs decreases and eventually drops to 0.

We now look at what happens when we fix *B*_*y*_ and vary the energy. Differing from the transmission profiles considered earlier, here we have at larger values of *B*_*y*_ a trend where the transmission at a given value of 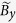 first drops to 0 as the energy is increased, and then increases from 0 as the energy is increased further. The region where the transmission is 0 essentially coincides with the energy band gap that opens up between the LEPB and HEHB.

**Figure 12:**
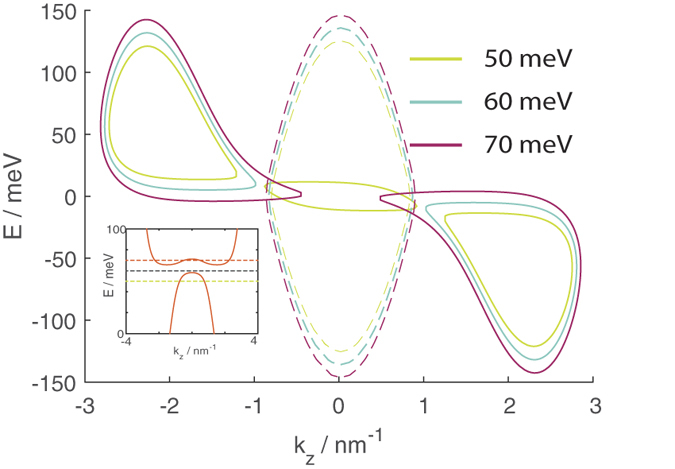
The EECs at 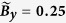 (solid lines) and 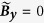 at the energies labeled on the legend. The inset shows the *k*_*z*_ dispersion relation for 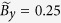 at *k*_*y*_ = 0 with the dotted horizontal lines indicating the energy levels at which the EECs are drawn.

This is also reflected in Fig. 12 which show the source and drain EECs at 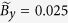, and *E* = 50, 60 and 70 eV. These energies correspond to those at the value of 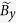 where the transmission decreases between 50 eV to 60 eV, then increases between 60 eV to 70 eV. The 50 eV drain EEC has a relative large *k*_*x*_ overlap with EEC due to the middle EEC curve enclosing |
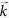
| = 0 which corresponds to the cross section of the HEPB. The next value of energy 60 eV corresponds to an energy where the *k*-space range enclosed by the source EEC falls in the gap between the HEHB and LEPB and the transmission is zero. The topmost value of energy is above the bottom of the LEPB where the EECs corresponding to the cross section of the LEPB increase in *k* space area and start to overlap with the source EEC again, giving rise to finite transmission.

## Conclusion

In this work we studied the effects of in-plane magnetic fields on a Na_3_Bi DSM thin film. We showed that in the absence of a magnetic field, there is a special band, the LEPB, which originates from the semi-infinite slab Fermi arcs which is localized near the surface of the thin films. The application of a magnetic field in the *y* direction leads to the sharpening of the LEPB band bottom and HEHB band top at 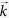 = 0, and a shifting of the energy where the LEPB and HEPB meet at 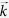 = 0 upwards when the field is first applied. As the field is increased further, the energy of the LEPB band at 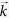 = 0 is shifted above that of the *k*-space surrounding and an energy gap opens between the LEPB and HEHB. Applying a magnetic field in the *z* direction leads to the broadening of the LEPB and the formation of an energy plateau along the *k* space *y* direction.

These changes affect the transmission from a source segment without a magnetic field to a drain segment with a magnetic field as the overlap in the *k*-space direction parallel to the interface between the source and drain EECs vary. In the particular case of an interface parallel to the *y* direction, the *k* space overlap is not very much affected by the magnetic field. The reduction in transmission by the magnetic field comes instead from the localization of the drain wavefunctions further inside the thickness of the film away from the source wavefunction localized near the film surface with increasing magnetic fields. The anisotropy between the *k* space direction parallel and perpendicular to the *k* space separation between the Weyl nodes give rise to differing transmission profiles for interfaces between the source and drain segments along different directions.

After the completion of this manuscript, we learnt of a very recent work^19^ on the effects of in-plane magnetic fields on Weyl and Dirac semimetals. In that work, the authors reasoned that an in-plane magnetic field applied parallel to the *k* space separation between the Weyl nodes (the *z* direction here) leads to a range of *k*_*y*_ perpendicular to the field in which there is no dispersion along the *k*_*z*_ direction. This agrees with our numerical results in Fig. 8a. Whereas ref. 19 did not explicitly study an in-plane field magnetic field with only components perpendicular to the Weyl node *k*-space separation, its authors did study an in-plane field applied at an angle to the *k*-space separation. Our results support their conclusion that the components of the in-plane field perpendicular to the *k*-space separation (*B*_*y*_) will lead to a shearing of the EEC along the *k*_*z*_ direction. Our EECs for a magnetic field in the *y* direction in Fig. 10 shows that *B*_*z*_ pushes the regions of the EEC with differing signs of *k*_*y*_ in opposite directions along the *k*_*z*_ direction. Our results show the additional feature that at large values of *B*_*z*_, this shearing causes the EEC to break up into separate closed curves.

## Additional Information

**How to cite this article**: Siu, Z. B. *et al*. Dirac semimetal thin films in in-plane magnetic fields. *Sci. Rep*. **6**, 34882; doi: 10.1038/srep34882 (2016).

## Supplementary Material

Supplementary Information
