# Supplementary material for: Dirac semimetal thin films in in-plane magnetic fields
Source: Sci Rep. 2016 Oct 10;6:34882. doi: 10.1038/srep34882 (PMC5056520; doi:10.1038/srep34882)
Supplement: Supplementary Information [file srep34882-s1.pdf]

Dirac semimetal thin films in in-plane magnetic fields

Supplementary Information

Zhuo Bin Siu [1*], Mansoor B.A. Jalil [1], and Seng Ghee Tan [2]
[1] Computational Nanoelectronics and Nanodevices Laboratory, National University of Singapore, Singapore [2] Data Storage Institute, Agency for Science, Reseach and Technology, Singapore
[*] Corresponding author : elesiuz@nus.edu.sg

I. CALCULATION OF THE FERMI ARCS

In this section we describe how we obtained the Fermi arcs for the semi-infinite slabs. Although we have arrived at our approach independently, we note that the calculation of the Fermi arcs has previously been reported [1, 2] before the completion of the manuscript.

Differing from an isotropic infinite bulk Na_3Bi which extends to $\pm\infty$ along all three of the x, y, z directions where the wavevectors $\vec{k}$ in $\exp(i\vec{k}\cdot\vec{r})$ of the Bloch states cannot contain imaginary parts, it is permissible for k_x to contain negative (positive) imaginary components for a semi-infinite slab extending to infinity along the y, z directions, and to semi-infinity from $x = 0$ to $x = -\infty$. ($x = +\infty$). These states with imaginary k_x components correspond to states localized near the boundary of the semi-infinite slab with the vacuum, and give rise to the Fermi arcs absent in the infinite bulk Na_3Bi as we shall now proceed to describe.

We consider a semi-infinite slab terminated at the x direction so that the slab extends to infinity along the y and z directions, and k_y and k_z are good quantum numbers. Adopting the hard-wall boundary condition where we demand that the wavefunction vanishes at $x = 0$, we look for linear combinations of the eigenstates of the spin up block of Eq. 1 in the main text which vanish at $x = 0$.

For the reader's convenience we reproduce the equations for the eigenenergies

$$\epsilon_\pm = \epsilon_0 \pm \sqrt{(Ak)^2 + M^2} \tag{S1}$$

and the (unnormalized) eigenspinors

$$|\chi\rangle = \begin{pmatrix} M \pm \sqrt{(Ak)^2 + M^2} \\ A(k_x - ik_y) \end{pmatrix} \tag{S2}$$

here.

In solving for k_x from the energy equation Eq. S1

$$\epsilon_{\pm_{(E)}} = \epsilon_0 \pm_{(E)} \sqrt{(Ak)^2 + M^2}$$

we shift the ϵ_0 to the LHS and square the resulting term, so the $\pm$ distinction in the square root is lost. Here, the subscript (E) indicates that this $\pm$ pertains to ϵ in order to distinguish it from the other $\pm$s that occur later.

In detail, we have

$$E = \epsilon_0 \pm \sqrt{(Ak)^2 + M^2}$$
$$\Rightarrow (E - \epsilon_0)^2 = (Ak)^2 + M^2$$

which gives a quadratic equation in k^2 (recall that ϵ_0 contains a k^2 term) so that we have, in turn, two values for k_x^2 for a given k_y. This quadratic equation is cumbersome and not particularly insightful and will not be presented here. We then obtain two values for k_x^2, which we designate as $k^2_{x;\pm_{(k_x^2)}}$. In general, only one of these roots will actually be the solution for a given $+/-$ sign value of the $\pm_{(E)}$.

Now seeking a linear superposition of eigenstates which disappear at the slab surface boundary, we examine the eigenspinors Eq. S2. Within the same $\pm_{(k_x^2)}$ branch, we have two values of $k_x = \pm_{(k_x)}\sqrt{k^2_{x;\pm_{(k_x^2)}}}$. If k_x were imaginary, then one of these will blow up in the wrong direction. If k_x is real, then the upper component of the eigenspinor has the same value for both signs of $\pm_{(k_x)}$, but the k_x in the lower component has opposite signs so it is impossible to form a linear combination of the two such that the eigenspinor vanishes. We therefore conclude that the linear superposition must consist of different $\pm_{(k_x^2)}$ branches.

Next, we investigate whether various quantities are real, complex or imaginary. E, k_z and k_y are given to be real. From $E = \epsilon_0 \pm \sqrt{(Ak)^2 + m^2}$ we conclude that ϵ_0 must be real because E is real and if ϵ_0 has any imaginary

component the imaginary component cannot be simultaneously canceled off by both signs of the square root term. ϵ_0 being real in turn restricts k_x^2 to be real, so k_x is either real or imaginary. E being real also constrains $\sqrt{(Ak)^2 + M^2}$ to be real. k^2 is real, so M^2 must be real as well. Thus the upper component of the eigenspinor is real. Earlier we noted that for the wavefunction to disappear at the boundary the two eigenspinors must come from different $\pm_{(k_x^2)}$ branches. We now know that the upper components of both branches are real, so the relative weights of the two eigenspinors are real. This in turn forces the k_x to be imaginary, so that the lower components of both eigenspinors are imaginary. (Assume that k_x is real so that the only imaginary part of the lower eigenspinor component comes from the ik_y portion. Then for the imaginary part of the lower eigenspinor component to cancel off we need the coefficients of the lower eigenspinor branches to be equal and opposite. This forces k_x to have the same value in both eigenspinors however, which gives the trivial solution of both eigenspinor components being 0.)

These insights allow us to suggest a numerical scheme to find the allowed values of (k_y, k_z) for a given value of energy E for a $\pm x$ terminated slab. An equation in k_y and k_z can be formed in the following way – for a given (k_y, k_z) we solve for the two values of k_x corresponding to the $\pm_{(k_x^2)}$ branches with the correct sign of the imaginary part depending on whether the semi-infinite slab is terminated along the $+x$ or $-x$ direction. Denoting these two values of k_x as $k_{x\pm_{(k_x^2)}}$, we back substitute them into Eq. S1 to determine which of the $\pm_{(E)}$ branches they correspond to, so that the correct form of the eigenspinors Eq. S2 can be obtained. With these two eigenspinors, which we denote as $|\pm_{(k_x)^2}\rangle$, we can then calculate the determinant of the linear equations in the unknown coefficients c_+ and c_- in $|+_{(k_x^2)}\rangle c_+ + |-_{(k_x^2)}\rangle c_- = 0$. The allowed values of (k_y, k_z) are then those for which the determinant vanishes. These values of (k_y, k_z) give the Fermi arcs.

II. THIN FILM EECS

We show in Supp. Fig. 1 the EECs and the number densities of the LEPB states at a few values of energy for a 50 nm thick film.

[1] Gorbar, E. V., Miransky, V. A., Shovkovy, I. A., and Sukhachov, P. O., Dirac semimetals A_3Bi (A = Na, K, Rb) as $\mathbb{Z}_2$ Weyl semimetals, *Phys. Rev. B* **91**, 121101(R) (2015).
[2] Gorbar, E. V., Miransky, V. A., Shovkovy, I. A., and Sukhachov, P. O., Surface Fermi arcs in $\mathbb{Z}_2$ Weyl semimetals A_3Bi (A=Na, K, Rb), *Phys. Rev. B* **91**, 235138 (2015).

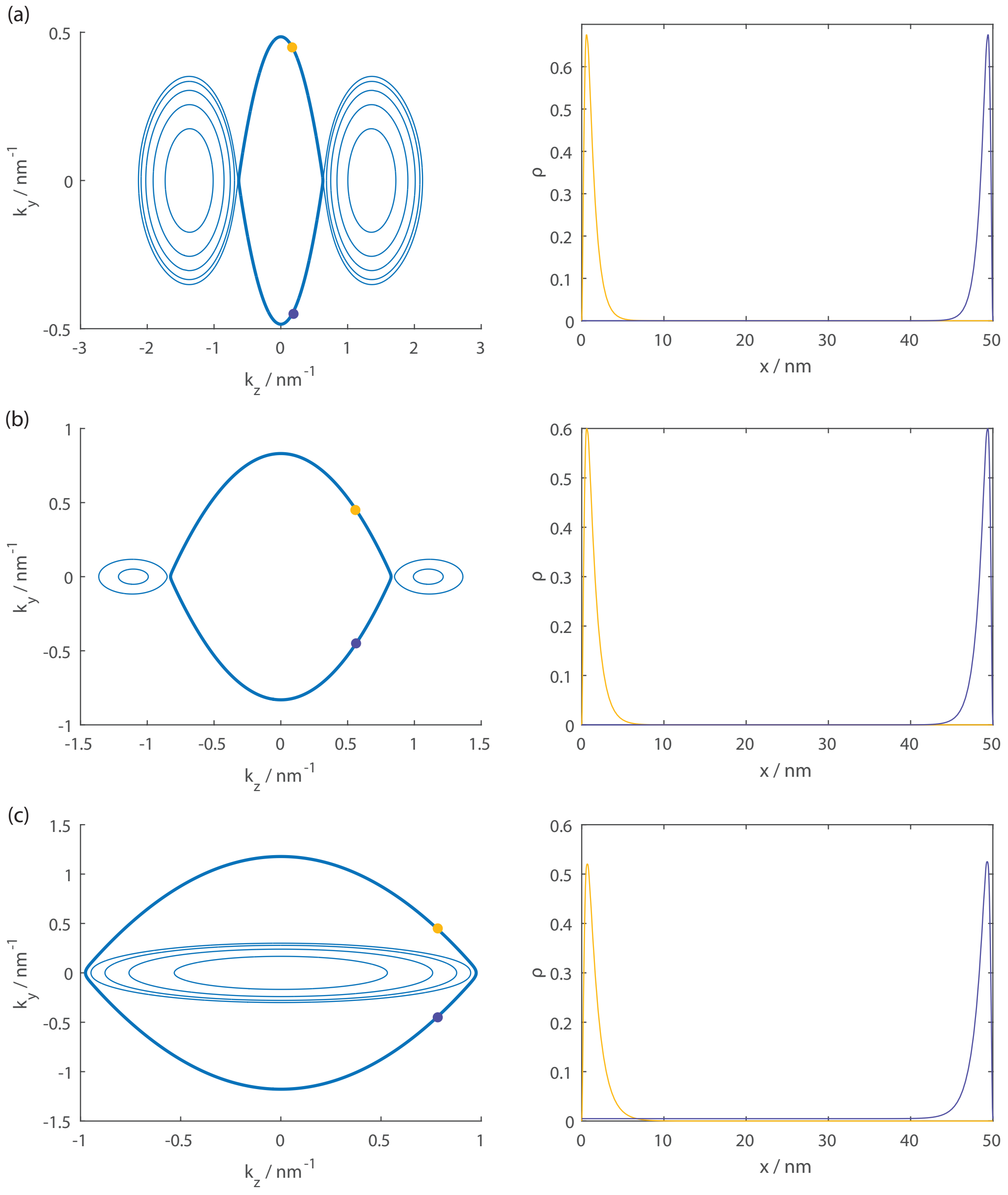

Supplementary Fig. 1: The EECs (left) and number densities (right) on the LEPB states (highlighted by thicker lines) at $k_y = \pm 0.4$ nm^{-1} for $E =$ (a) 0 meV, (b) 50 meV and (c) 100 meV for a 50 nm thick film.
